# Predation of newly settled Dungeness crab by juvenile sunflower sea stars

**DOI:** 10.1002/ecy.70379

**Published:** 2026-04-07

**Authors:** Miles E. Rough, Aaron W. E. Galloway

**Affiliations:** ^1^ Oregon Institute of Marine Biology University of Oregon Charleston Oregon USA

**Keywords:** Dungeness crabs, kelp forest ecology, *Metacarcinus magister*, predation, *Pycnopodia helianthoides*, sunflower sea stars, top‐down control, trophic subsidies

Sunflower sea stars (*Pycnopodia helianthoides*) are voracious, generalist predators that consume a wide variety of benthic invertebrates across intertidal and subtidal zones (Duggins, 1983; Shivji, 1983; Sloan & Robinson, 1983). Historically abundant from the northern Aleutian Island chain in Alaska to Baja, California, populations of *P. helianthoides* have declined precipitously (>94% across the range) following the outbreak of sea star wasting disease in 2013 (Hamilton et al., 2021). *P. helianthoides* is currently listed as Critically Endangered (IUCN, 2020) with estimated population losses numbering in the billions (Hamilton et al., 2021; IUCN, 2020).

As one of the largest and fastest sea stars, *P. helianthoides* are capable of actively pursuing and capturing highly mobile prey, a trait that distinguishes them from many other sea stars. This capacity for active predation mirrors patterns seen in kelp forest consumers, which often derive substantial portions of their diet from both pelagic and benthic sources (Elliott Smith et al., 2021). While numerous studies have documented *P. helianthoides* trophic interactions and ecological roles (Burt et al., 2018; Galloway et al., 2023; Shivji, 1983), previous work has focused on adult stars, and the diets of juvenile *P. helianthoides*, and the consumption of pelagic‐sourced production in general by this key predator remains unexplored. Here, we define “juvenile” *P. helianthoides* as stars with a radius of <10 cm, following Hodin et al. (2021).

In June 2025, during a collection dive for *P. helianthoides* in Port Orford, Oregon, USA, we observed dense aggregations of recently settled Dungeness crab (*Metacarcinus magister*) juveniles, as previously described by Galloway et al. (2017). These recruits, based on their size, were likely settled from their life as pelagic zooplankton megalopae within days to weeks of this observation. At the time of the observation, the juvenile crabs were primarily concentrated in small sand pockets, between the rocky reef habitats where *P. helianthoides* were collected.

Fourteen juvenile *P. helianthoides* (4.2–7.9 cm radius) were collected from Port Orford, Oregon, USA, on 1 June and 3 June 2025 from two locations: Port Orford Jetty (42.737689 N, −124.498104 W) and Tichenor Cove (42.738672 N, −124.504046 W). Dense aggregations of newly settled *M. magister* recruits were present at both sites, but no direct predation by *P. helianthoides* was observed while underwater. Once *P. helianthoides* were collected, they were placed in a cooler and transported to the Oregon Institute of Marine Biology, Charleston, Oregon, USA, which served as a temporary holding prior to their transfer to the Oregon Coast Aquarium in Newport, Oregon, USA, where they were going to be held for future experiments and ultimately released, as per our ODFW permits (no. 28855 [*M. magister*] and no. 28880 [*P. helianthoides*]). Once the *P. helianthoides* were removed from the cooler and placed into the holding tanks, multiple *M. magister* recruits were observed at the bottom of the cooler, including one live individual. As predation on juvenile crab by *P. helianthoides* has not been previously reported, this observation represents a novel trophic interaction.

*P. helianthoides* were transported to Oregon Coast Aquarium, Newport, USA, on 4 June 2025. After sizing, weighing, and measuring each individual, they were separated into individual tanks and given four *M. magister* recruits (approximately 0.40–1.10 cm carapace width). Once *M. magister* individuals were captured by the *P. helianthoides*, a new crab was added to maintain a constant density of four *M. magister* per tank. The feeding trial lasted approximately 47 h, during which the 14 *P. helianthoides* individuals consumed a total of 145 *M. magister* recruits (Figures 1b and 2). This corresponds to an average of 10.4 recruits per individual over the trial, or roughly 5.2 per day. Consumption rates varied among individuals, ranging from 4 recruits (~2 per day) to 16 recruits (~8 per day) (Appendix S1: Table S1). This variation was associated with the radius of *P. helianthoides*, as prey consumption increased significantly with body size (Poisson GLM: β = 0.187, *p* = 0.025). Although the recruits were markedly faster than *P. helianthoides*, the sea stars were able to capture them by extending their arms to corner the prey and seizing them with tube feet or pedicellariae, small, pincer‐like structures on the aboral side (Figure 1a).

**FIGURE 1:**
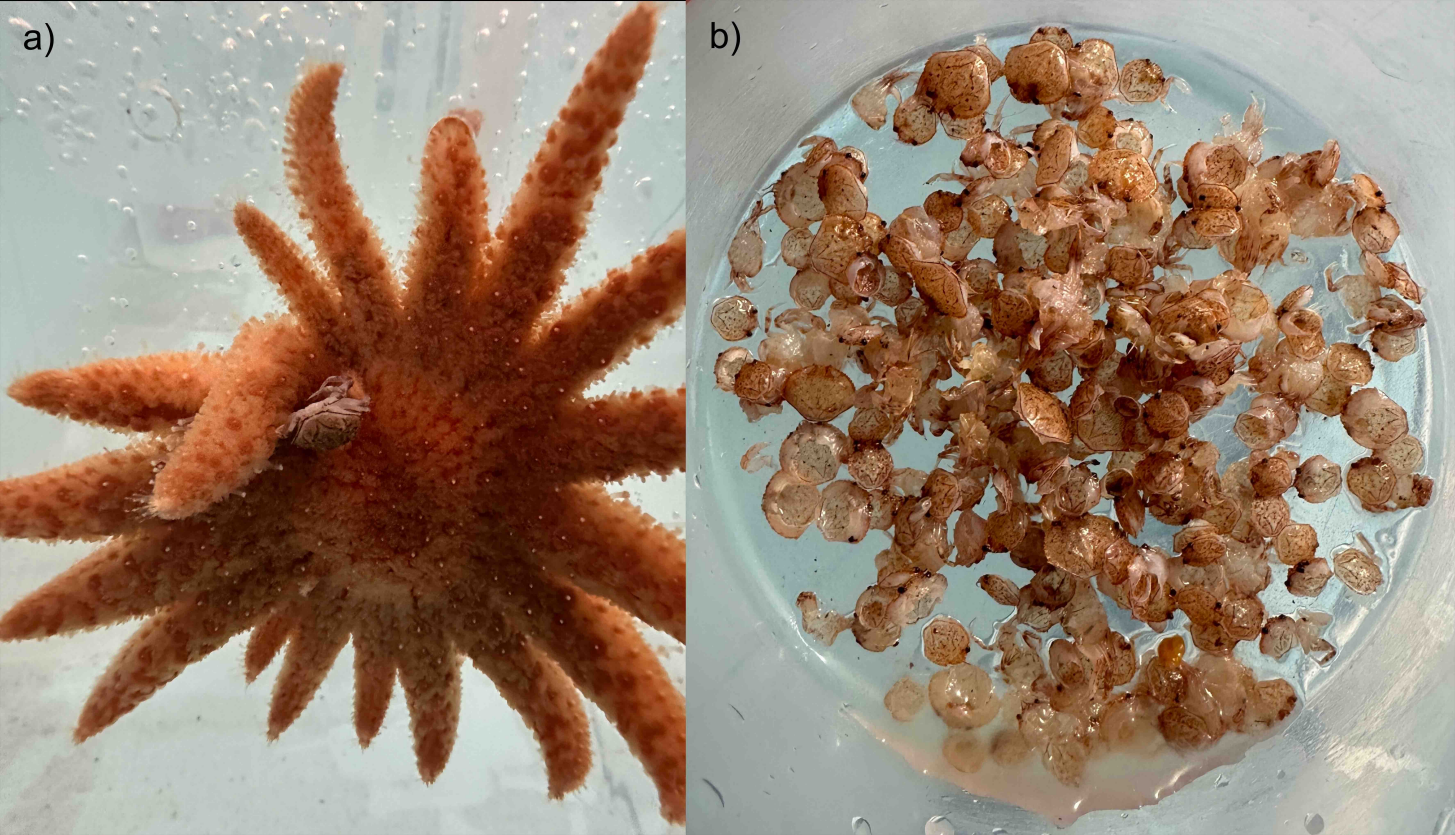
(a) *Metacarcinus magister* getting stuck on the pedicellariae of *Pycnopodia helianthoides* allowing it to be caught before it was eventually brought down to the oral side and consumed. (b) *M. magister* carapaces taken out of tanks at the Oregon Coast Aquarium during the entirety of the 47 h feeding trial. Photos by Miles E. Rough, June 2025.

**FIGURE 2:**
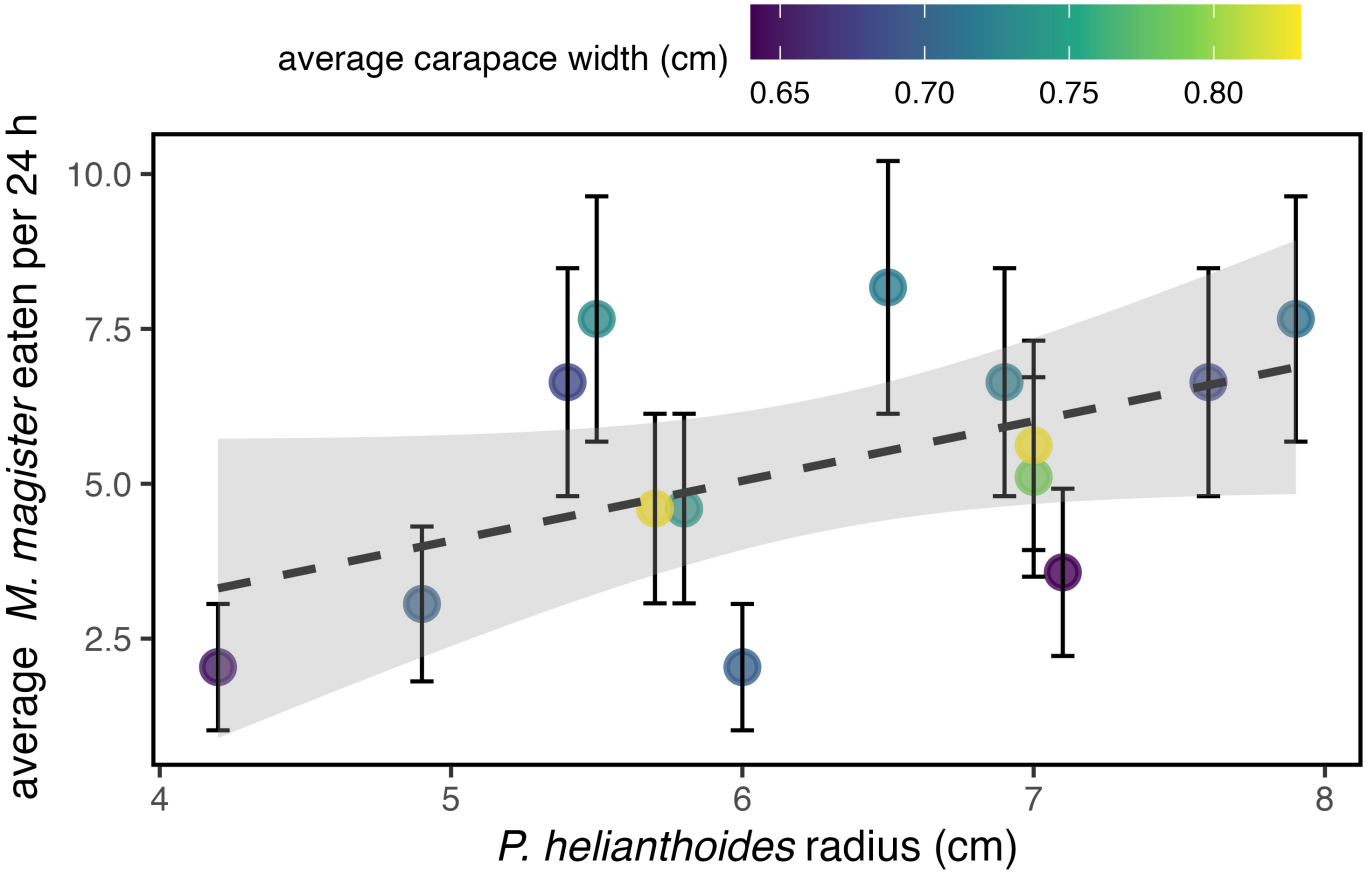
Average number of *Metacarcinus magister* consumed per 24 h by individual *Pycnopodia helianthoides* of different sizes. Points represent individual *P. helianthoides*, colored by average carapace length of *M. magister* consumed. Vertical error bars indicate the expected SD under a Poisson distribution. The gray dashed line shows a linear regression fit, and the gray shaded area indicates the 95% CI of the regression. Prey consumption increased significantly with star radius (Poisson GLM: β = 0.187, *p* = 0.025).

Our observation captures a previously unreported but potentially ecologically consequential moment where a benthic predator directly consumes newly settled recruits soon after they have arrived from the plankton, providing a clear example of cross‐ecosystem subsidies which link pelagic‐derived prey to nearshore benthic food webs (e.g., Zuercher & Galloway, 2019). Like benthivorous reef fishes, which can derive up to 98% of their diet from pelagic sources (Docmac et al., 2017), *P. helianthoides* may similarly access recently settled crab recruits as a highly concentrated energy input during the juvenile life stage for both organisms.

These observations and feeding trials provide the first documented evidence that juvenile *P. helianthoides* actively consumes recently settled *M. magister*. *P. helianthoides* is known to sometimes consume various adult “unknown” crab species (Duggins, 1983; Herrlinger, 1983; Mauzey et al., 1968; Shivji, 1983). There are also a few observations of adult *P. helianthoides* eating *n* = 1 Cancer crabs, including *Cancer oregonensis* (Paul & Feder, 1975), *Cancer productus* (Appendix S1: Figure S1, Galloway unpublished, Herrlinger, 1983), and *M. magister* (Feder & Hoberg, 1980). However, crabs are generally rare in stomach content analyses, ranging from >1% to 10% of the identified prey in these studies. While *P. helianthoides* is a known generalist predator and scavenger with broad diets, meaningful predation on early life stages of decapod crustaceans has not been previously reported, except for an observation of one megalopa of an unknown crab species found in one *P. helianthoides* in Central California (Herrlinger, 1983). The finding here that juvenile *P. helianthoides* can eat ~5 juvenile crabs per day suggests that this predator could exert direct top‐down control on crabs soon after their benthic settlement and recruitment, potentially influencing population dynamics of *M. magister* at local scales.

## AUTHOR CONTRIBUTIONS

**Miles E. Rough:** Conceptualization; data curation; formal analysis; investigation; methodology; writing original draft; writing review and editing. **Aaron W. E. Galloway:** Resources; supervision; formal analysis; writing original draft; writing review and editing.

## FUNDING INFORMATION

This work was partially supported by the Oregon Kelp Alliance (NOAA Restoration Center Cooperative Agreement NA25NMFX469C0012) and The Nature Conservancy (P119034) awarded to Aaron W.E. Galloway.

## CONFLICT OF INTEREST STATEMENT

The authors declare no conflicts of interest.

## Supporting information


Appendix S1.

## Data Availability

Data (Rough & Galloway, 2026) are available in Figshare at https://doi.org/10.6084/m9.figshare.31403556.v2.

## References

[ecy70379-bib-0001] Burt, J. M. , M. T. Tinker , D. K. Okamoto , K. W. Demes , K. Holmes , and A. K. Salomon . 2018. “Sudden Collapse of a Mesopredator Reveals its Complementary Role in Mediating Rocky Reef Regime Shifts.” Proceedings of the Royal Society B: Biological Sciences 285: 20180553. 10.1098/rspb.2018.0553 PMC6083256 30051864

[ecy70379-bib-0002] Docmac, F. , M. Araya , I. A. Hinojosa , C. Dorador , and C. Harrod . 2017. “Habitat Coupling Writ Large: Pelagic‐Derived Materials Fuel Benthivorous Macroalgal Reef Fishes in an Upwelling Zone.” Ecology 98: 2267–2272. 28632943 10.1002/ecy.1936

[ecy70379-bib-0003] Duggins, D. O. 1983. “Starfish Predation and the Creation of Mosaic Patterns in a Kelp‐Dominated Community.” Ecology 64: 1610–1619.

[ecy70379-bib-0004] Elliott Smith, E. A. , C. Harrod , F. Docmac , and S. D. Newsome . 2021. “Intraspecific Variation and Energy Channel Coupling within a Chilean Kelp Forest.” Ecology 102: e03198. 33009678 10.1002/ecy.3198

[ecy70379-bib-0005] Feder, H. M. , and M. K. Hoberg . 1980. The Epifauna of Three Bays (Port Etches, Zaikof Bay and Rocky Bay) in Prince William Sound, Alaska, with Notes on Feeding Biology 1–40. Fairbanks, AK: University of Alaska, Institute of Marine Science.

[ecy70379-bib-0006] Galloway, A. W. E. , S. A. Gravem , J. N. Kobelt , W. N. Heady , D. K. Okamoto , D. M. Sivitilli , V. R. Saccomanno , J. Hodin , and R. Whippo . 2023. “Sunflower Sea Star Predation on Urchins Can Facilitate Kelp Forest Recovery.” Proceedings of the Royal Society B: Biological Sciences 290: 20221897. 10.1098/rspb.2022.1897 PMC9943640 36809801

[ecy70379-bib-0007] Galloway, A. W. E. , A. L. Shanks , S. Groth , S. R. Marion , and A. R. Thurber . 2017. “Massive Crab Recruitment Events to the Shallow Subtidal Zone.” Ecology 98: 1468–1470. 28418594 10.1002/ecy.1740

[ecy70379-bib-0008] Hamilton, S. L. , V. R. Saccomanno , W. N. Heady , A. L. Gehman , S. I. Lonhart , R. Beas‐Luna , F. T. Francis , et al. 2021. “Disease‐Driven Mass Mortality Event Leads to Widespread Extirpation and Variable Recovery Potential of a Marine Predator across the Eastern Pacific.” Proceedings of the Royal Society B: Biological Sciences 288: 20211195. 10.1098/rspb.2021.1195 PMC8385337 34428964

[ecy70379-bib-0009] Herrlinger, T. J. 1983. “The Diet and Predator‐Prey relationships of the Sea Star *Pycnopodia Helianthoides* (Brandt) from a Central California Kelp forest.” Moss Landing Marine Laboratories San Jose State University.

[ecy70379-bib-0010] Hodin, J. , A. Pearson‐Lund , F. P. Anteau , P. Kitaeff , and S. Cefalu . 2021. “Progress toward Complete Life‐Cycle Culturing of the Endangered Sunflower Star, *Pycnopodia helianthoides* .” The Biological Bulletin 241: 243–258. 35015622 10.1086/716552

[ecy70379-bib-0011] IUCN . 2020. “*Pycnopodia helianthoides*: Gravem, S.A., Heady, W. N., Saccomanno, V. R., Alvstad, K. F., Gehman, A. L. M., Frierson, T. N. & Hamilton, S.L.” The IUCN Red List of Threatened Species 2021. e.T178290276A197818455.

[ecy70379-bib-0012] Mauzey, K. P. , C. Birkeland , and P. K. Dayton . 1968. “Feeding Behavior of Asteroids and Escape Responses of their Prey in the Puget Sound Region.” Ecology 49: 603–619.

[ecy70379-bib-0013] Paul, A. J. , and H. M. Feder . 1975. “The Food of the Sea Star *Pycnopodia helianthoides* (Brandt) in Prince William Sound, Alaska.” Ophelia 14: 15–22.

[ecy70379-bib-0014] Rough, M. , and A. Galloway . 2026. “Code and Raw Data for Analyses from Predation of Newly Settled Dungeness Crab by Juvenile Sunflower Sea Stars.” Figshare. 10.6084/m9.figshare.31403556.v2 PMC13054779 41944081

[ecy70379-bib-0015] Shivji, M. 1983. “Feeding and Distribution Study of the Sunflower Sea Star *Pycnopodia helianthoides* (Brandt, 1835).” Pacific Science 37: 133–140.

[ecy70379-bib-0016] Sloan, N. A. , and S. M. C. Robinson . 1983. “Winter Feeding by Asteroids on a Subtidal Sandbed in British Columbia.” Ophelia 22: 125–140.

[ecy70379-bib-0017] Zuercher, R. , and A. W. E. Galloway . 2019. “Coastal Marine Ecosystem Connectivity: Pelagic Ocean to Kelp Forest Subsidies.” Ecosphere 10: e02602.

