# Supplementary material for: Predation of newly settled Dungeness crab by juvenile sunflower sea stars
Source: Ecology. 2026 Apr 7;107(4):e70379. doi: 10.1002/ecy.70379 (PMC13054779; doi:10.1002/ecy.70379)
Supplement: Supplementary file 1 — Appendix S1. [file ECY-107-e70379-s001.pdf]

Predation of newly settled Dungeness crab by juvenile sunflower sea stars

Miles E. Rough and Aaron W.E. Galloway

Ecology

Appendix S1

Table S1. Summary of feeding activity of individual *P. helianthoides* over 47 hours. Total number of *M. magister* consumed, average number of prey consumed per day (mean +/- SD) and average carapace length (cm, mean +/- SD) are shown for the fourteen individuals collected on 3-June and 1-June 2025 from Port Orford Jetty and Tichenor Rock, Port Orford, Oregon, USA at ~10 m depth.

***P. helianthoides* radius (cm)**	**total *M. magister* eaten**	**average eaten per 24 hr ± SD**	**average carpus length (cm) ± SD**
7	10	5.11 ± 1.61	0.79 ± 0.24
7	11	5.62 ± 1.69	0.83 ± 0.17
7.9	15	7.66 ± 1.98	0.71 ± 0.09
6.5	16	8.17 ± 2.04	0.72 ± 0.10
6.9	13	6.64 ± 1.84	0.72 ± 0.14
5.8	9	4.60 ± 1.53	0.74 ± 0.18
4.9	6	3.06 ± 1.25	0.70 ± 0.17
5.5	15	7.66 ± 1.98	0.73 ± 0.13
7.1	7	3.57 ± 1.35	0.64 ± 0.17
6	4	2.04 ± 1.02	0.70 ± 0.20
5.4	13	6.64 ± 1.84	0.68 ± 0.16
5.7	9	4.60 ± 1.53	0.83 ± 0.16
4.2	4	2.04 ± 1.02	0.65 ± 0.06
7.6	13	6.64 ± 1.84	0.68 ± 0.11

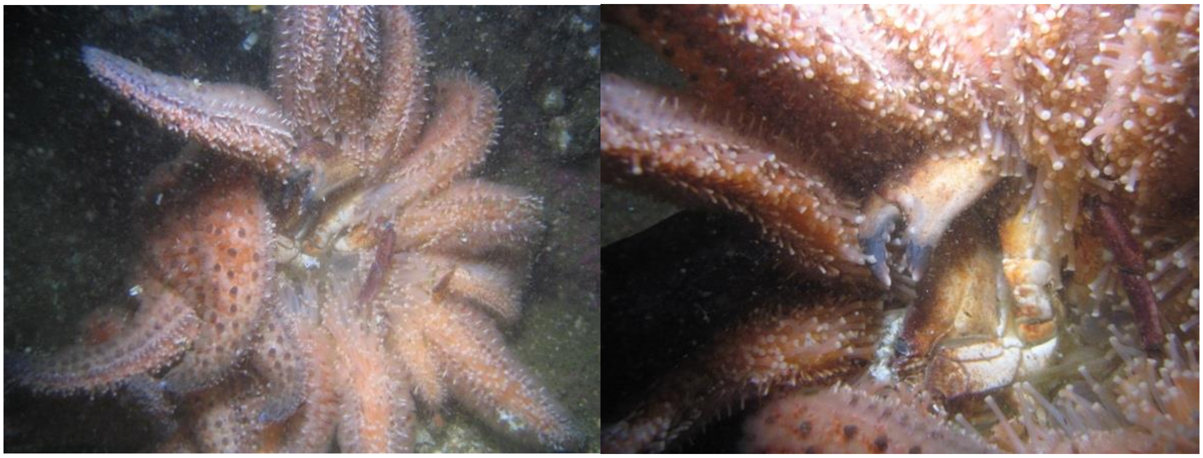

Figure S1. Photographs of an adult (radius ~35 cm) *P. helianthoides* eating an adult red rock crab (*Cancer productus*) in Puget Sound, Washington (Sunrise Park, near Gig Harbor: N 47.347663, W -122.555605). The sunflower sea star was found humped up (indication of eating) and partially dug into a sand substrate, having ambushed the buried crab, which was alive and still slightly moving when found (i.e., the crab is not a molt). Photos by Aaron W.E. Galloway, June 2007.
